# Human mesenchymal stromal cells inhibit *Mycobacterium avium* replication in clinically relevant models of lung infection

**DOI:** 10.1136/thorax-2023-220819

**Published:** 2024-03-20

**Authors:** Timothy D Shaw, Anna D Krasnodembskaya, Gunnar N Schroeder, Declan F Doherty, Johnatas Dutra Silva, Shikha M Tandel, Yue Su, David Butler, Rebecca J Ingram, Cecilia M O'Kane

**Affiliations:** Wellcome-Wolfson Institute for Experimental Medicine, Queen's University, Belfast, UK

**Keywords:** Atypical Mycobacterial Infection, Macrophage Biology, Respiratory Infection, Innate Immunity, Opportunist lung infections

## Abstract

**Introduction:**

Novel therapeutic strategies are urgently needed for *Mycobacterium avium* complex pulmonary disease (MAC-PD). Human mesenchymal stromal cells (MSCs) can directly inhibit MAC growth, but their effect on intracellular bacilli is unknown. We investigated the ability of human MSCs to reduce bacterial replication and inflammation in MAC-infected macrophages and in a murine model of MAC-PD.

**Methods:**

Human monocyte-derived macrophages (MDMs) were infected with *M. avium* Chester strain and treated with human bone marrow-derived MSCs. Intracellular and extracellular colony-forming units (CFUs) were counted at 72 hours. Six-week-old female balb/c mice were infected by nebulisation of *M. avium* Chester. Mice were treated with 1×10^6^ intravenous human MSCs or saline control at 21 and 28 days post-infection. Lungs, liver and spleen were harvested 42 days post-infection for bacterial counts. Cytokines were quantified by ELISA.

**Results:**

MSCs reduced intracellular bacteria in MDMs over 72 hours (median 35% reduction, p=0.027). MSC treatment increased extracellular concentrations of prostaglandin E2 (PGE2) (median 10.1-fold rise, p=0.002) and reduced tumour necrosis factor-α (median 28% reduction, p=0.025). Blocking MSC PGE2 production by cyclo-oxygenase-2 (COX-2) inhibition with celecoxib abrogated the antimicrobial effect, while this was restored by adding exogenous PGE2. MSC-treated mice had lower pulmonary CFUs (median 18% reduction, p=0.012), but no significant change in spleen or liver CFUs compared with controls.

**Conclusion:**

MSCs can modulate inflammation and reduce intracellular *M. avium* growth in human macrophages via COX-2/PGE2 signalling and inhibit pulmonary bacterial replication in a murine model of chronic MAC-PD.

**WHAT IS ALREADY KNOWN ON THIS TOPIC**

- Novel therapeutic strategies are urgently needed for the growing problem of *Mycobacterium avium* complex pulmonary disease (MAC-PD). Human mesenchymal stromal cells (MSCs) are under investigation as a host-directed therapy for infectious and inflammatory lung conditions, but little is known about their potential in MAC-PD.

**WHAT THIS STUDY ADDS**

- Human MSCs modulate inflammation and reduce intracellular MAC replication in primary human macrophages and reduce pulmonary bacterial burden in an animal model of chronic MAC-PD.

**HOW THIS STUDY MIGHT AFFECT RESEARCH, PRACTICE OR POLICY**

- These findings warrant further investigation into human MSCs as an adjunctive, host-directed therapy for MAC-PD.

## Introduction

*Mycobacterium avium* complex (MAC) is an emerging, multidrug-resistant pathogen driving a global rise in pulmonary disease (MAC-PD).^1^ In susceptible patients, MAC establishes an intracellular replicative niche in lung macrophages and promotes progressive inflammatory tissue damage.^2^ MAC is multidrug resistant and current treatment regimens consist of protracted courses of poorly tolerated antimicrobial combinations.^3^ There is an urgent need for novel strategies to target intracellular MAC, shorten antimicrobial regimens and attenuate inflammatory lung damage.

Mesenchymal stromal cells (MSCs) are non-haematopoietic multipotent adult stromal cells that contribute to tissue regeneration.^4^ As an intravenous administration, MSCs are typically captured in the lung microvasculature and home to sites of inflammation.^5^ They undergo Toll-like receptor activation and secrete antimicrobial peptides with broad-spectrum activity.^6^ MSCs recruit circulating monocytes, promote their differentiation into tissue macrophages,^7^ augment their phagocytotic ability and enhance intracellular bacterial killing via mitochondrial transfer,^8^ efferocytosis,^9^ extracellular vesicles^10^ and paracrine factors.^11^ MSCs also regulate excessive inflammation and reduce tissue damage through modulating dendritic cells,^12^ natural killer cells^13^ and lymphocytes.^14^

The simultaneous enhancement of immune-pathogen clearance and inflammatory regulation has attracted interest in MSC therapies for infectious and inflammatory lung diseases including tuberculosis (TB),^15^ community-acquired pneumonia,^16^ chronic obstructive pulmonary disorder (COPD)^17^ and cystic fibrosis.^18^ To date, only one preclinical report has tested human MSCs against MAC,^19^ finding they had direct antimicrobial activity in vitro and reduced pulmonary MAC burden in a 7-day cystic fibrosis mouse model (Cftr^−/−^) infected by transtracheal infusion of bacterial-loaded beads. This short study provided preliminary evidence for MSC antimicrobial activity against MAC but did not explore mechanism of efficacy in vitro nor their effect on chronic MAC-PD in vivo.

We investigated the ability of human MSCs to inhibit bacterial replication and reduce inflammation in clinically relevant models of MAC-PD: (1) an in vitro model of intracellular MAC infection using primary human macrophages and (2) a mouse model of chronic MAC-PD.

## Methods

Additional details are available in the online supplemental file.

10.1136/thorax-2023-220819.supp1 Supplementary data

### Bacterial culture

*M. avium* subsp *avium* Chester ATCC 25291 (American Type Culture Collection) reference strain was cultured in Middlebrook 7H9 and quantified by counting colony-forming units per mL (CFUs/mL) on Middlebrook 7H11 as previously described.^20^ Where specified, a clinical strain isolated from sputum of a patient with clinically confirmed *M. avium* pulmonary disease (*M. avium* CI5) was used in confirmation studies (donated by the Northern Ireland Mycobacterial Reference Laboratory).

### Human monocyte-derived macrophage isolation

Monocytes were isolated from blood donor residual buffy coat or peripheral blood by density centrifugation across a Ficoll-Paque gradient (Sigma-Aldrich), followed by adherence, selection and differentiation into monocyte-derived macrophages (MDMs) as previously described.^21 22^ MDMs were differentiated into alveolar-like macrophages using granulocyte macrophage colony-stimulating factor (GM-CSF) (Peprotech) to more closely model MAC infection of alveolar macrophages.^23 24^ These MDMs have been previously confirmed to express M1-like markers (CD40+ and CD54+) with relative absence of M2-like markers (CD163+ and CD206+) on flow cytometry.^25^

### MSC culture

Human bone marrow-derived MSCs (BM-MSCs) from ATCC (PCS-500-012) were cultured in α-Minimal Essential Medium supplemented with 16.5% heat-inactivated fetal bovine serum, 1% L-glutamine and 100 µg/mL ampicillin (Thermo Fisher). For confirmation studies, human BM-MSCs from a second source were obtained from the Texas A&M Health Science Centre College of Medicine, Institute for Regenerative Medicine, USA as previously described.^8^ MSCs at passages 2–6 were added to infected MDMs either directly or in transwells (0.4 µm diameter pores; Merck Millipore) at a ratio of 1 MSC:3 MDMs. Human pulmonary fibroblasts (CCD-11Lu cells, ATCC) were used as a stromal cell control.

### Cellular infection studies

MDMs were infected with MAC at multiplicity of infection (MOI) of 1 for 4 hours before washing and replacing media. Cell lysates and supernatants were collected as previously described.^20^

### Quantification of immune-signalling molecules

Human interleukin (IL)-6, IL-8, interferon (IFN)-γ, tumour necrosis factor (TNF)-α, IDO, GM-CSF, KGF and murine IL-6, CXCL-1 and TNF-α were quantified using ELISA DuoSet kits (R&D Systems) according to the manufacturer’s instructions. Human IL-1β, IL-18 and IL-10 were quantified using Luminex Multiplex Assay (Bio-teche) according to the manufacturer’s instructions. Prostaglandin E2 (PGE2) was quantified by parameter assay (R&D Systems). Nitric oxide (NO) was quantified in cell supernatants using a Griess Reagent Kit (Thermo Fisher), according to the manufacturer’s instructions.

### Animal studies

All experiments were performed in accordance with Animal Research: Reporting of In vivo Experiments guidelines. Six-week-old female balb/c mice (Charles River) were exposed to nebulised *M. avium* in a plethysmography double-chamber (EMMS) at 50% airspace density for 5 min. Mice were randomised to receive either 1 million human BM-MSCs in 100 µL saline or vehicle control via tail vein injection on days 21 and 28 post-infection. On day 42 post-infection, whole lungs, spleen and liver were harvested for CFU and protein quantification.

### Statistical analysis

Power calculations were performed a priori to guide sample size for in vitro and in vivo studies (see online supplemental data). Data were analysed using GraphPad Prism V.9 software. Unless otherwise stated, the Mann-Whitney U test was used for comparison of two experimental groups and the Kruskal-Wallis test with Dunn’s multiple comparisons was performed for three or more experimental groups. For comparison of experimental groups with additional variables (eg, changes over time), a two-way analysis of variance model was used.

## Results

### MSCs inhibit intracellular replication of MAC in infected MDMs

**Figure 1:**
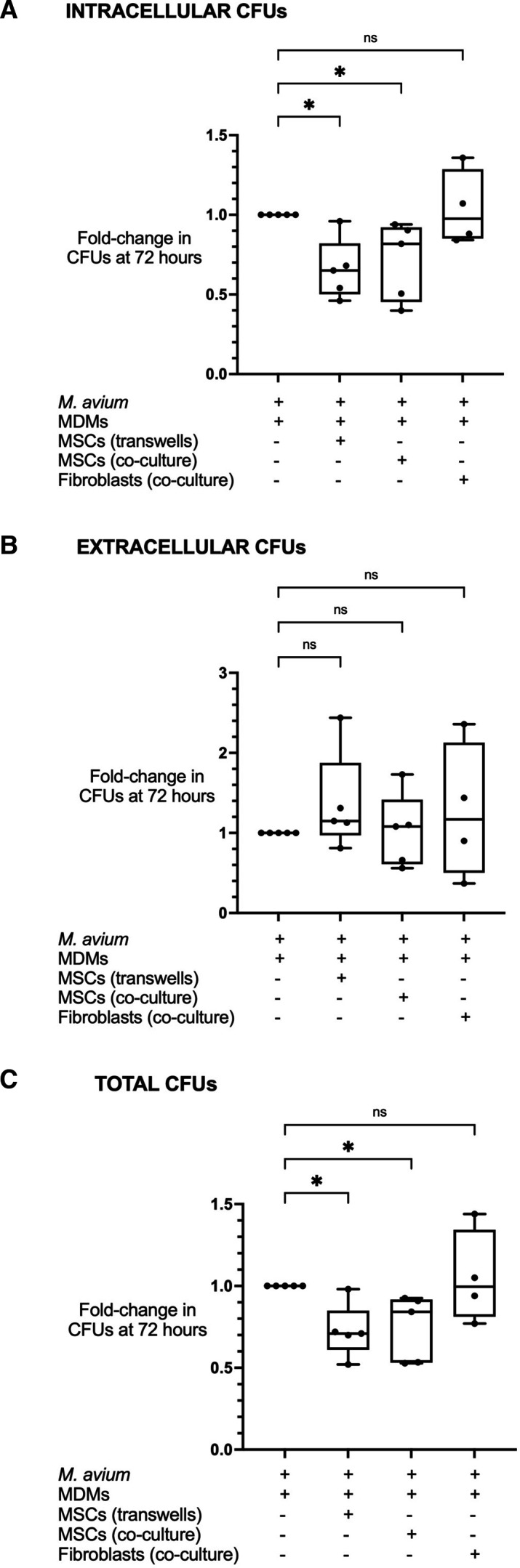
The effect of MSCs in an in vitro MDM infection model. There was a significant fall in intracellular CFUs in MDMs treated with MSCs in transwells and co-culture (p<0.05) (A). Pulmonary fibroblasts did not affect intracellular CFUs. There was no significant change in extracellular CFUs following either treatment (B). Combining intracellular and extracellular CFUs, treatment with MSCs, but not pulmonary fibroblasts, mediated a significant fall in total CFUs in transwells and in co-culture (both p<0.05) (C). All conditions included MDMs infected with *Mycobacterium avium* for 4 hours before washing, with + signs referring to treatments added after washing. Data presented as median with IQR and analysed using the Kruskal-Wallis test with Dunn’s multiple comparison test. N=4–5 (pulmonary fibroblasts were not available for one of five experimental replicates which proceeded without them to avoid wastage of valuable primary MDMs and MSCs in time-sensitive studies). *P<0.05. CFUs, colony-forming units; MDMs, monocyte-derived macrophages; MSCs, mesenchymal stromal cells; ns, not significant.

MAC-infected MDMs were treated with MSCs in transwells or co-culture for 72 hours to assess the role of paracrine and cell contact mechanisms, respectively. Intracellular CFUs fell in MDMs treated with MSCs in transwells (median 0.35-fold reduction, p=0.027) and MSCs in co-culture (median 0.18-fold change, p=0.044) (figure 1A). In contrast, fibroblasts had no effect on intracellular CFUs. Extracellular CFUs were not significantly changed by MSCs or by fibroblasts (figure 1B).

At 72 hours post-infection, intracellular CFUs were approximately one log-fold higher than extracellular CFUs for each MDM donor, indicating that the bacterial burden in these assays was predominantly intracellular (online supplemental table 1). Total CFUs fell by median 0.29-fold (p=0.023) and 0.16-fold (p=0.044) in MDMs treated with MSCs in transwells or co-culture, respectively, but were unchanged in fibroblast-treated MDMs (figure 1C).

We repeated experiments using the clinical isolate *M. avium* CI5 from a patient with MAC-PD (online supplemental figure 1A) and with human BM-MSCs isolated from a different source (online supplemental figure 1B), confirming a similar pattern of inhibited intracellular replication, indicating the effect was neither strain nor MSC-donor specific.

To test whether MSCs directly inhibited growth of MAC (independent of macrophages), bacteria were cultured in the presence of MSCs at MOI of 3, either separated by a transwell or in direct co-culture. There was no significant difference in bacterial growth over 72 hours in co-culture with MSCs compared with media control (online supplemental figure 2), suggesting that these MSCs did not have direct antimicrobial activity against MAC.

### MSCs specifically modulate PGE2 and TNF-α levels during MDM infection

**Figure 2:**
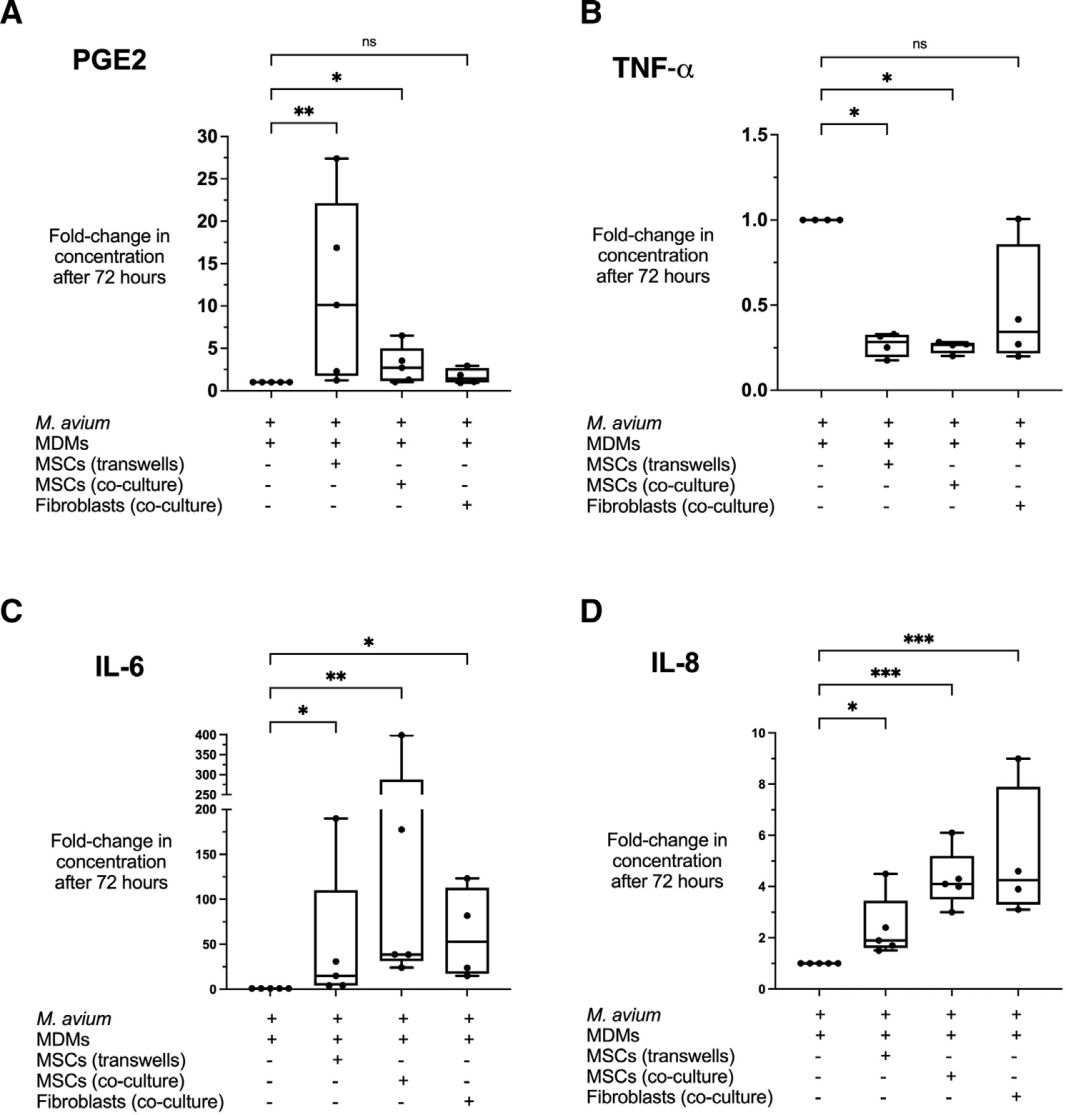
Modulation of cytokine by MSCs during *Mycobacterium avium* infection of MDMs. Treatment of infected MDMs with MSCs, but not with fibroblasts, resulted in elevated PGE2 (A) and reduced TNF-α (B) (both p<0.05). Levels of IL-6 (C) and IL-8 (D) also rose in MSC-treated conditions, but this was also observed with fibroblast treatment. All conditions included MDMs infected with *M. avium* for 4 hours before washing, with + signs referring to treatments added after washing. Data presented as median with IQR and analysed using the Kruskal-Wallis test with Dunn’s multiple comparison test. N=4–5 (pulmonary fibroblasts were not available for one of five experimental replicates which proceeded without them to avoid wastage of valuable primary MDMs and MSCs in time-sensitive studies). *P<0.05; **p<0.01; ***p<0.001. IL, interleukin; MDMs, monocyte-derived macrophages; MSCs, mesenchymal stromal cells; ns, not significant; PGE2, prostaglandin E2; TNF-α, tumour necrosis factor-α.

The inhibitory effect of MSCs on intracellular MAC was independent of cell contact with macrophages, so we investigated the role of soluble mediators known to be important in host response to mycobacterial infection: PGE2, TNF-α, IL-6, IL-8, IL-1β, IL-18, IL-10, IFN-γ, IDO, GM-CSF and NO.^26^ Concentrations of these mediators were measured in the supernatants of infected MDMs±treatments in the experimental series using the ATCC source of human BM-MSCs. PGE2 concentrations were significantly elevated in the supernatant of MAC-infected MDMs treated with MSCs in transwells (median 10.1-fold, p=0.002) (figure 2A). MSCs in co-culture mediated a modest rise in PGE2 (median 2.7-fold, p<0.05). In contrast, TNF-α concentrations were significantly decreased when infected MDMs were treated with MSCs, whether in transwells (median 0.72-fold reduction, p=0.020) or co-culture (median 0.73-fold reduction, p=0.025) (figure 2B). Fibroblasts did not significantly affect PGE2 or TNF-α concentrations.

MSCs also mediated a rise in IL-6 and IL-8, though this was also observed with fibroblast treatment (figure 2C,D). NO levels were below the level of detection in infected MDM supernatants, even after MSC treatment (data not shown). No significant change was observed for IL-1β, IL-18, IL-10, IFN-γ, IDO and GM-CSF after treatment with MSCs (online supplemental figure 3).^26^

### The inhibitory effect of MSCs is cyclo-oxygenase-2 dependent and mediated by PGE2

**Figure 3:**
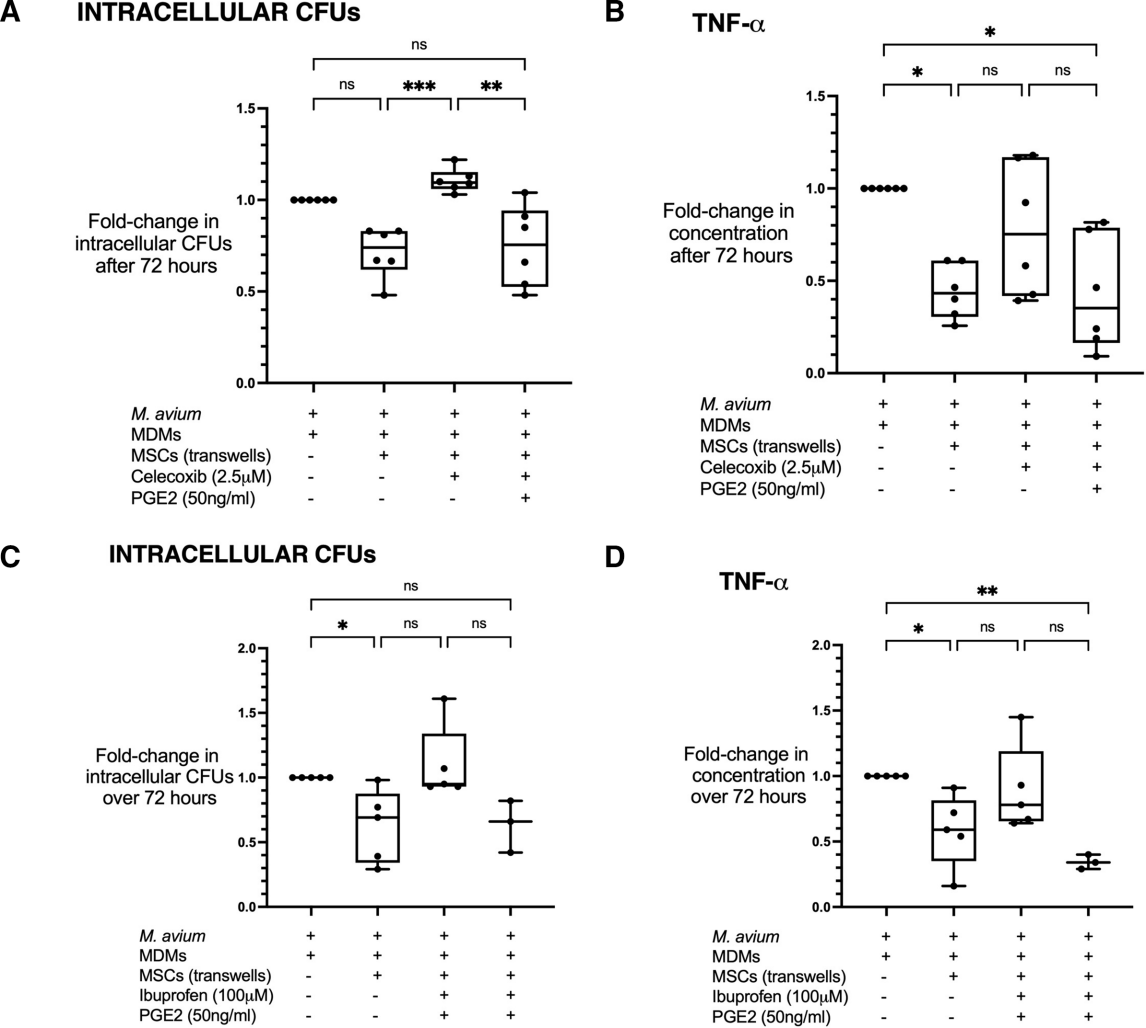
The role of COX/PGE2/PI3K signalling in MSC modulation of *Mycobacterium avium*-infected MDMs. The fall in intracellular CFU mediated by MSC treatment in transwells was abrogated by the addition of celecoxib (p<0.001) but rescued by the addition of exogenous PGE2 (p<0.01) (A). A similar pattern was observed for MSC modulation of TNF-α (B). Similarly, ibuprofen (a non-selective COX inhibitor) abrogated the fall in intracellular CFU (C) and TNF-α concentrations (D) in MSC-treated MDMs, which was rescued by the addition of exogenous PGE2 (p<0.05 and p<0.01, respectively). All conditions included MDMs infected with *M. avium* for 4 hours before washing, with + signs referring to treatments added after washing. Data presented as median with IQR and analysed using the Kruskal-Wallis test with Dunn’s multiple comparison test. N=6 for A+B; N=3–5 for C+D (PGE2 treatments not given beyond three experimental replicates to preserve MDMs as effect demonstrated with tight error bars). *P<0.05; **p<0.01; ***p<0.001. CFUs, colony-forming units; COX, cyclo-oxygenase; MDMs, monocyte-derived macrophages; MSCs, mesenchymal stromal cells; ns, not significant; PGE2, prostaglandin E2; PI3K, phosphoinositide 3-kinase; TNF-α, tumour necrosis factor-α.

Following these results, we investigated whether PGE2 secretion was important for inhibition of intracellular MAC. Cyclo-oxygenase-2 (COX-2) is a critical enzyme for the synthesis of PGE2 upon inflammatory stimuli.^27^ We hypothesised that if PGE2 was responsible for the antimicrobial effect of MSCs, then COX-2 inhibition would abrogate it. First, we established that PGE2 concentrations rose when MAC-infected MDMs were treated with MSCs, but not with MSC-conditioned media (consisting of constitutive secretome of unstimulated MSCs collected after 24-hour culture and filtered to remove cells) (online supplemental figure 4A). This confirmed that the PGE2 response was associated with cell–cell interaction between MSCs and MAC-infected MDMs. Next, MSCs in transwells were treated with the COX-2-specific inhibitor celecoxib (2.5 µM) for 30 min. Transwells were then inserted above infected MDMs and celecoxib was added to maintain 2.5 µM concentration. Celecoxib treatment abrogated the antimicrobial effect of MSCs in MAC-infected MDMs (figure 3A), and we confirmed it inhibited MSC-induced PGE2 production (online supplemental figure 4B). Addition of exogenous PGE2 50 ng/mL (Sigma-Aldrich) restored the inhibitory effect (median 0.24-fold reduction, p=0.004).

MSC-induced reduction in TNF-α was also COX-2 dependent and restored by addition of exogenous PGE2 (figure 3B), demonstrating that this was a downstream consequence of PGE2 signalling. Similar results were observed when MSCs were treated with the non-selective COX inhibitor ibuprofen 100 µM (Sigma-Aldrich) with or without exogenous PGE2 (figure 3C,D).

**Figure 4:**
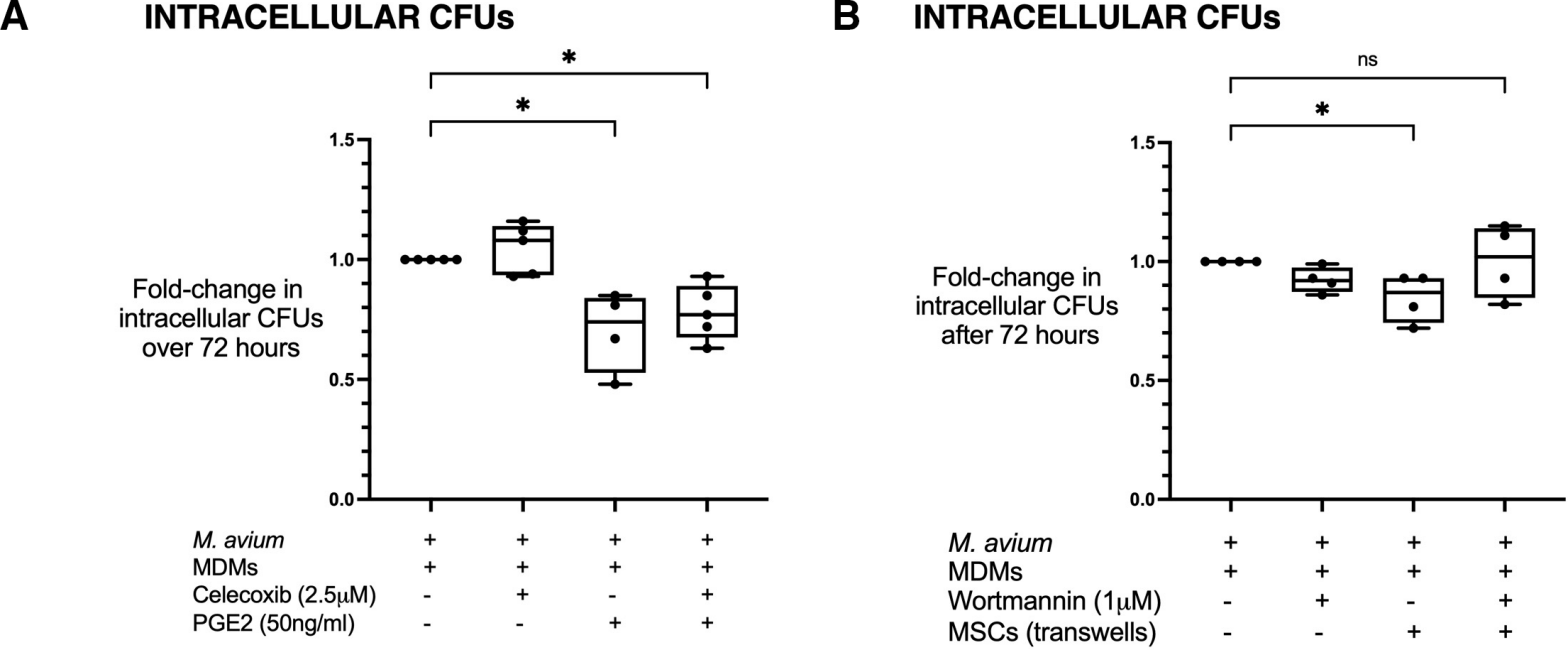
MSC modulates MAC-infected macrophages via the COX-2/PGE2/PI3K pathway. Celecoxib alone did not significantly affect intracellular CFUs, but the inhibitory effect observed with MSC treatment could also be replicated in their absence by addition of PGE2 (p<0.05) (A). After pretreating infected MDMs with the PI3K inhibitor wortmannin, MSCs no longer induced a significant fall in intracellular bacteria (B). All conditions included MDMs infected with *Mycobacterium avium* for 4 hours before washing, with + signs referring to treatments added after washing. Data presented as median with IQR and analysed using the Kruskal-Wallis test with Dunn’s multiple comparison test. N=4–5. *P<0.05. CFUs, colony-forming units; COX-2, cyclo-oxygenase-2; MAC, *Mycobacterium avium* complex; MDMs, monocyte-derived macrophages; MSCs, mesenchymal stromal cells; ns, not significant; PGE2, prostaglandin E2; PI3K, phosphoinositide 3-kinase.

We expected the celecoxib would diffuse through the well and eventually come to have inhibitory activity on MDM COX-2. Therefore, we sought to investigate the effect of COX-2 inhibition in infected MDMs and their subsequent response to exogenous PGE2 treatment. To elucidate whether celecoxib or PGE2 had any direct effect on intracellular growth independently of MSCs, infected MDMs were treated with celecoxib and/or PGE2 only. Celecoxib alone had no significant effect on intracellular CFUs after 72 hours but PGE2 alone reduced intracellular CFUs by median 0.26-fold reduction (p<0.05) (figure 4A). This antibacterial effect was not affected by inhibiting macrophage COX-2 with celecoxib, indicating that COX-2-dependent production of PGE2 in MSCs and its paracrine effect on infected macrophages were responsible for inhibiting intracellular MAC replication.

### PGE2 inhibits intracellular bacterial growth by activating phosphoinositide 3-kinase in infected MDMs

PGE2 binds to membrane-bound E-prostanoid receptors on macrophages to activate a cascade of intracellular signalling pathways,^27^ including phosphoinositide 3-kinase (PI3K) which promotes phagolysosomal maturation and lysosomal degradation via phosphorylation of AKT.^28^ We investigated whether the effect of MSC-derived PGE2 on intracellular bacterial replication was PI3K dependent.

First, we characterised the timing of MSC activation and PGE2 secretion when suspended in transwells above infected MDMs. PGE2 concentrations were measured at several time points from 30 min to 48 hours after addition of the MSCs in transwells to infected MDMs from three different donors (online supplemental figure 4C). Interdonor (MDM) variation in the PGE2 secretion from MSCs was observed, particularly in the first 4 hours post-treatment. By 8 hours, PGE2 concentration had reached approximately 1000 pg/mL for all three MDM donors and continued to rise more than 10-fold over 48 hours. We found macrophage AKT was phosphorylated progressively over 24 hours after infection with MAC in the presence of MSCs (online supplemental figure 4D) confirming activation of the AKT pathway. We then used the selective, irreversible inhibitor of PI3K wortmannin (Tocris), to disrupt macrophage PI3K signalling.^29^ MAC-infected MDMs were pretreated with wortmannin 1 µM for 30 min before washing and treatment with MSCs in transwells. Wortmannin inhibited the effect of MSCs on intracellular bacteria (figure 4B). Taken together, these findings suggest that inhibition of intracellular replication of MAC in infected macrophages by MSCs is mediated by COX-2-dependent production, secretion of PGE2 and activation of macrophage PI3K.

### MSCs reduce MAC burden during chronic pulmonary infection in mice

**Figure 5:**
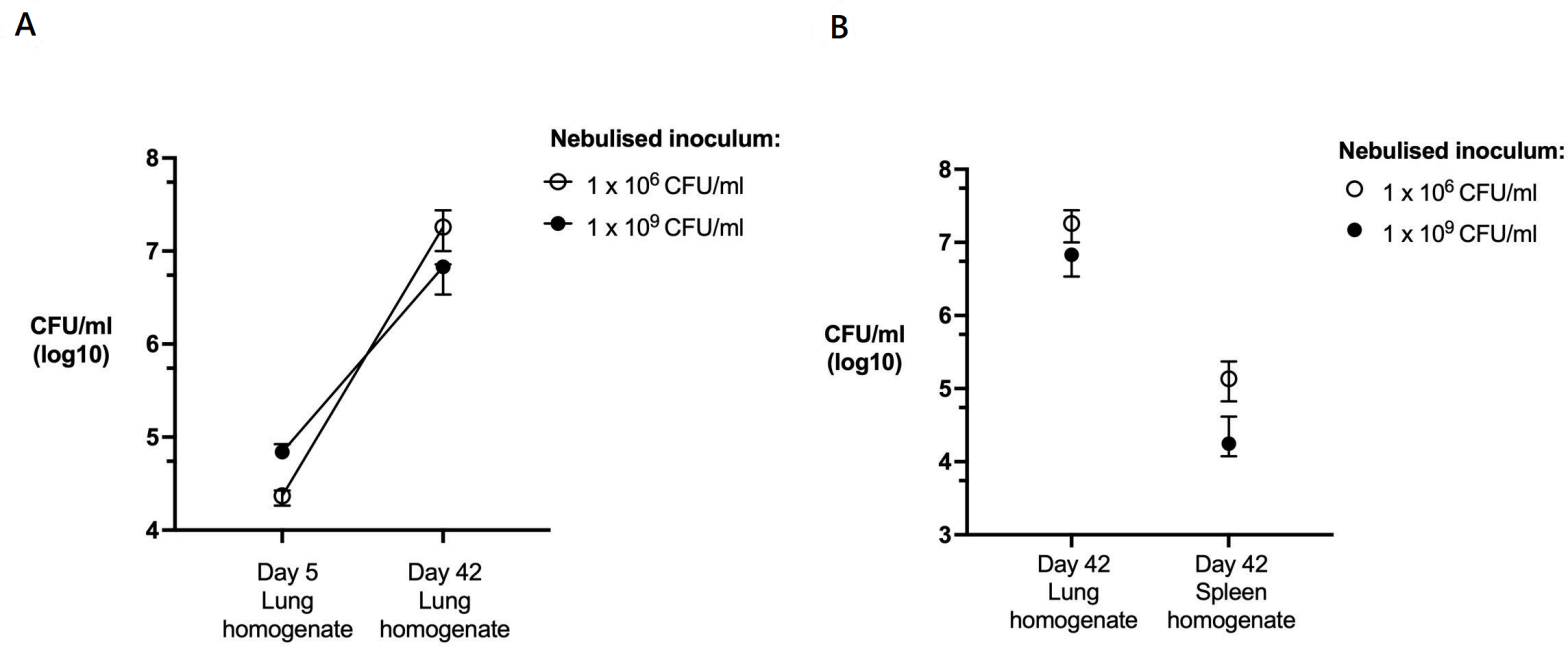
MAC-PD is generated by exposure to infectious aerosols. The burden of pulmonary infection at day 5 post-infection correlated with the concentration of nebulised solution and there was significant proliferation of pulmonary infection between days 5 and 42 (p<0.01) (A) which resulted in dissemination to spleen (B). Data presented as median with IQR, and groups were analysed by two-way analysis of variance for inoculum and time. N=5–10 per time point. CFU, colony-forming unit; MAC-PD, *Mycobacterium avium* complex pulmonary disease.

**Figure 6:**
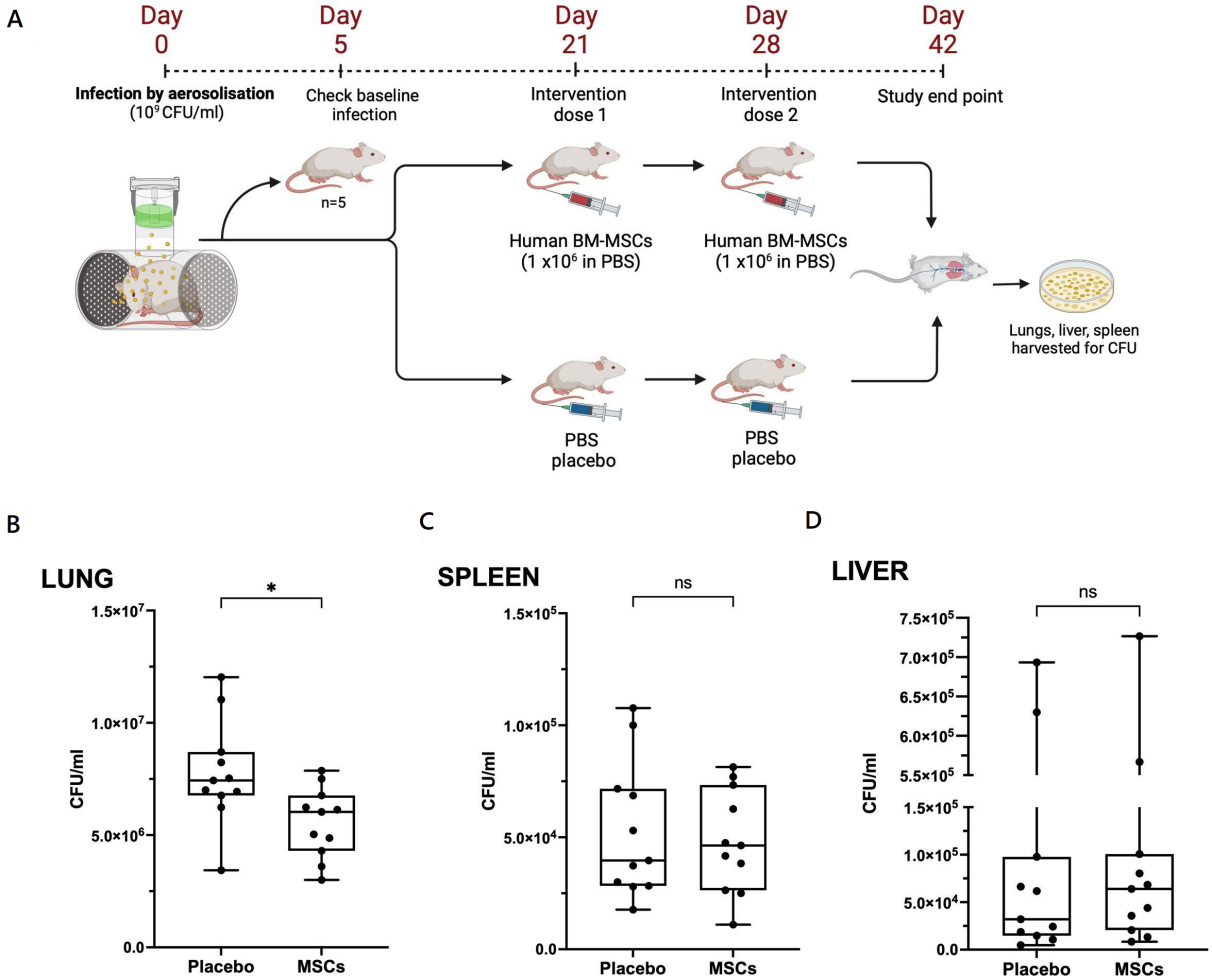
MSCs reduce lung bacterial burden in mice with chronic *Mycobacterium avium* pulmonary disease. Female 6-week-old balb/c mice were exposed to nebulised *M. avium* (10^9^ CFU/mL) and baseline infection was confirmed at 5 days post-infection (A). Mice were treated with intravenous injection of 1 million human BM-MSCs or placebo on days 21 and 28 post-infection and culled at day 42 post-infection to assess for organ CFUs and protein quantification. MSC-treated mice had reduced pulmonary CFUs at day 42 compared with placebo group (p<0.05) (B). However, CFU counts were comparable between MSC and placebo groups for spleen (C) and liver (D). Data presented as median with IQR, and groups were analysed by Mann-Whitney U test. N=11 mice per group and data combined from two independent experiments. *P<0.05. BM-MSCs, bone marrow-derived MSCs; CFUs, colony-forming units; MSCs, mesenchymal stromal cells; ns, not significant; PBS, phosphate-buffered saline. Image (A) created using Biorender.

**Figure 7:**
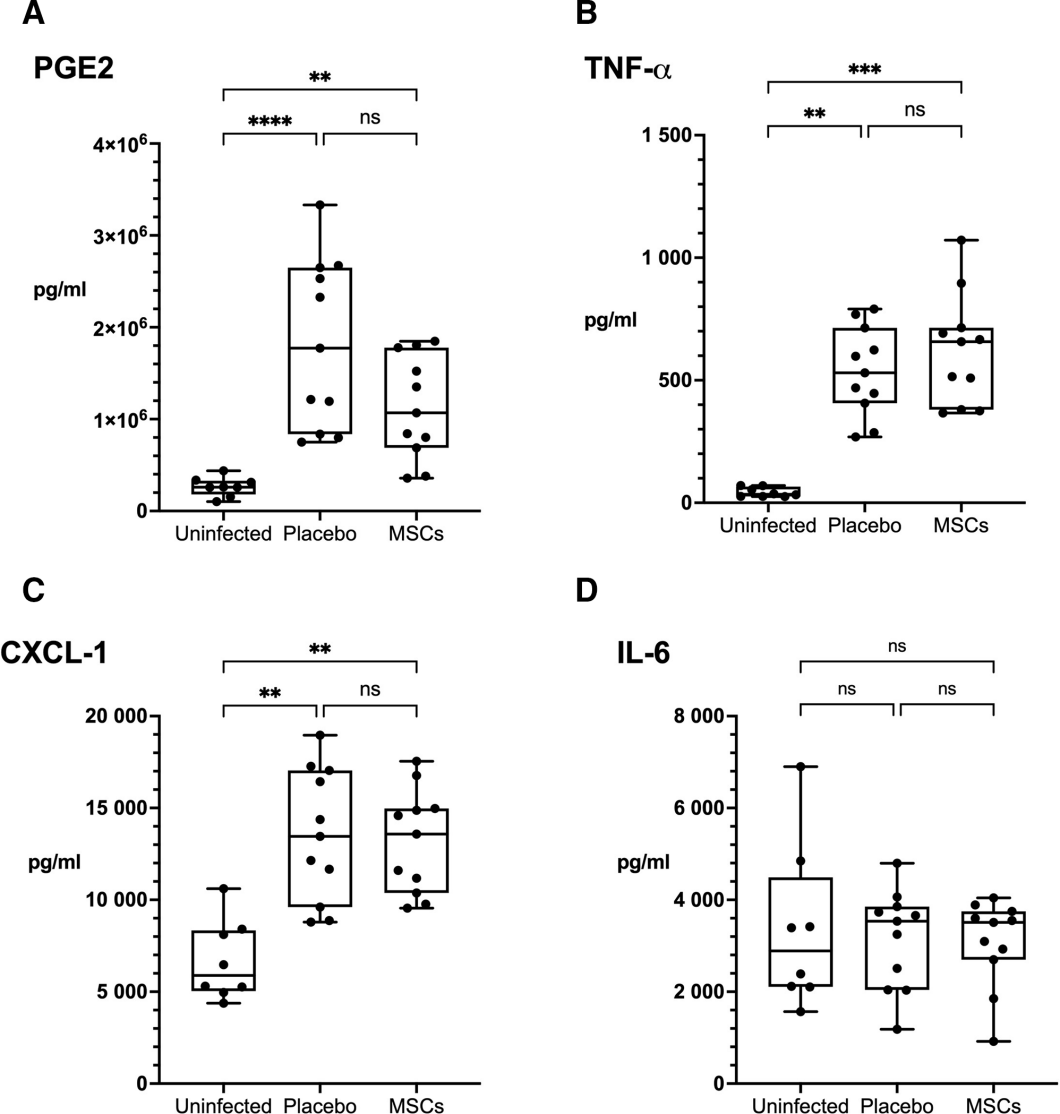
The effect of MSC treatment on pulmonary mediators of inflammation. After 42 days, mice with chronic pulmonary *Mycobacterium avium* infection had significantly increased levels of PGE2 (A), TNF-α (B) and CXCL-1 (C) levels in the clarified lung homogenate compared with uninfected mice (p<0.001, p<0.01 and p<0.01, respectively), but IL-6 concentrations were comparable between groups (D). MSC treatment did not significantly change cytokine or PGE2 levels compared with placebo. Data presented as median with IQR, and groups were analysed by Kruskal-Wallis test with Dunn’s multiple comparison test. N=8 (uninfected) or 11 (placebo and MSC-treated) and data combined from two independent experiments. *P<0.05; **p<0.01; ***p<0.001; ****p<0.0001. IL, interleukin; MSCs, mesenchymal stromal cells; ns, not significant; PGE2, prostaglandin E2; TNF-α, tumour necrosis factor-α.

These data suggest that MSCs enhance macrophage inhibition of intracellular MAC replication. To assess the activity in vivo, we investigated the effect of MSCs on a murine model of chronic pulmonary MAC infection. We showed that a single 5-minute exposure to nebulised MAC established a proliferative pulmonary infection with extrapulmonary dissemination over 42 days (figure 5). We exposed mice to nebulised *M. avium* (10^9^ CFU/mL), which resulted in a median lung infection at day 5 of 1.1×10^5^ CFU (IQR 7.0×10^4^–2.1×10^5^, n=10) (online supplemental table 2). Mice were randomised to receive either 1 million human BM-MSCs or control (saline) at days 21 and 28 post-infection via tail vein injection (figure 6A). At day 42, lung bacterial burden in the placebo-treated group had risen to median 7.4×10^6^ CFU, confirming bacterial replication. MSC-treated mice had a significant reduction in pulmonary bacteria compared with placebo group (median 6.0×10^6^ CFU vs 7.4×10^6^ CFU per lung, p=0.012) (figure 6B). There was no difference in splenic or liver CFUs between groups (figure 6C,D). Mice with pulmonary MAC infection had significantly increased lung concentration of PGE2 (p<0.001), TNF-α (p<0.01) and CXCL-1 (p<0.01) compared with uninfected mice (figure 7). IL-6 concentrations were comparable between infected and uninfected mice. There was no significant difference in cytokine concentrations between MSC-treated and placebo groups. Uninfected mice gained more weight than infected mice treated with placebo or MSCs over the 42-day experiment, though this did not reach statistical significance (online supplemental figure 5A). There was also no significant difference in weight gain between placebo-treated and MSC-treated mice (online supplemental figure 5B).

## Discussion

The immunomodulatory properties of MSCs are well-known, with much interest around their therapeutic potential in inflammatory disorders.^7^ More recently, evidence of their broad and potent antimicrobial activity has emerged,^30 31^ but their potential in mycobacterial infection remains to be established.^26^ We found that human MSCs inhibit intracellular replication of MAC and modulate the inflammatory response in primary human macrophages. These effects are dependent on MSC COX-2 activity and mediated by secretion of PGE2 which acts on macrophages via a PI3K-dependent pathway. Likewise, two doses of human MSCs reduced lung bacterial burden in mice with chronic MAC-PD.

Clinically relevant modelling of chronic MAC infection remains underdeveloped and hinders preclinical screening of candidate therapies. This study builds on work by Bonfield *et al* which found that a single dose of human MSCs reduced lung CFUs of MAC after 7 days in Cftr^−/−^ mice infected with surgically implanted beads.^19^ In our study, lung infection was generated in immunocompetent balb/c mice using infectious aerosols which more closely imitates a whole lung infection. We modelled MAC-PD using Chester strain which has been validated for in vivo antimicrobial testing in balb/c mice^32^ and administered MSCs in the chronic phase (weeks 3–4 vs 24 hours post-infection) which better resembles the clinical scenario of treating established disease. The 18% reduction in pulmonary CFUs upon MSC treatment in our study was modest compared with log-fold fall previously reported but is comparable with 2 weeks of standard antimicrobial regimens (rifampicin, ethambutol and clarithromycin) which achieved 20% reduction in chronic murine MAC-PD with Chester strain.^33^ The discrepancy in efficacy between studies may be attributable to the higher bacterial burden and accompanying pathology in chronic disease, as well as variable responsiveness to therapies between the mouse strains. Recurring MSC treatments, up to four doses, have been required to detect efficacy in other settings (such as a fall in C reactive protein (CRP) in patients with COPD^17^), reflecting their rapid clearance in vivo. Repeat dosing of MSCs is now well established and tolerated in clinical trials.^26^ The lack of antimicrobial effect of MSCs on established infection in the liver and spleen may relate to MSCs being largely trapped in the pulmonary microvasculature or lung, where they may undergo apoptosis or efferocytosis by lung macrophages,^9^ resulting in migration of only small numbers of MSCs to distal sites.^5^ The effect on lung macrophages would be most clinically relevant since systemic infection with MAC is rare outside profound immunocompromise, for example, in advanced HIV infection.^3^

MSCs inhibited intracellular MAC replication in primary human macrophages by median 35% over 72 hours, which compares favourably with standard antimicrobial treatments (rifampicin, ethambutol and clarithromycin) given to macrophages infected with the same strain and duration.^33^ We found MSCs had no direct activity against planktonic MAC in vitro, in contrast to Bonfield *et al* who reported human BM-MSCs in co-culture reduced CFUs of MAC by approximately 45% over 72 hours.^19^ However, they found considerable source-dependent heterogeneity and MSCs from 2 of 12 sources displayed no detectable activity against MAC, consistent with the MSCs from our sources. This may be related to differential secretion of antimicrobial peptides^34^ and the intrinsic resistance of MAC to some AMPs secreted by MSCs.^35^ We also found interdonor variation in macrophage response to MSC treatments, with some demonstrating greater intracellular control of MAC than others. Interdonor variation in biological responses between primary human cells is a well-recognised challenge, attributable partly to differences in gene expression and protein function,^36^ with many factors remaining poorly understood.

We found MSC-secreted PGE2 to be the key mediator of their immunomodulatory effect in vitro, as reported in infection studies elsewhere.^7 11^ In comparison, other inflammatory mediators were not significantly changed (such as IDO and GM-CSF) or not specifically modulated by MSCs (such as IL-6 and IL-8 which were also increased by fibroblast cell control). MSC-secreted products have been investigated as an alternative to whole cell therapy for some conditions, on account of pragmatic advantages (greater resilience through freeze–thaw cycles), safety concerns (reduced risk of replication or engraftment) and economic benefits (less costly to manufacture, store and transplant).^26^ However, we found the in vitro PGE2 response was associated with whole MSC treatment only and was not generated by treating infected MDMs with MSC-conditioned media. We suggest this is because the therapeutic mechanism of MSCs (COX-2 activation and PGE2 secretion) is dependent upon activation at the site of infection, which cannot be replicated with constitutively produced MSC products.

PGE2 is protective in early *M. tuberculosis* infection where it activates macrophage PI3K, promoting phagolysosomal fusion and bacterial killing.^37^ Mutations affecting EP2 receptor and PGE synthase function increase susceptibility to TB in mice and humans.^38 39^ However, COX inhibition appears host protective in established TB where increased PGE2 impairs adaptive immunity.^40^ The importance of COX activity in MAC-PD is not yet known. We found COX-2 inhibition of MDMs did not affect their permissiveness of intracellular bacterial growth, whereas exogenous PGE2 treatment reduced intracellular bacteria and TNF-α concentration in MDMs. However, PGE2 and TNF-α concentrations were unchanged in the lungs of MSC-treated mice, despite a fall in bacterial counts. We suggest that any signal of inflammatory modulation may have been transient and undetectable by the study endpoint as exogenous MSCs are typically cleared within 72 hours.^41^ Alternatively, MSCs may exert host-protective effects in vivo through means other than, or in addition to, PGE2 secretion.^10^

MSC treatments have been investigated in other mycobacterial infections, including *M. tuberculosis* and *M. abscessus*, though reported outcomes are mixed.^42^ Murine BM-MSCs improved bacterial clearance in mice with pulmonary *M. abscessus* infection after 10 days, associated with increased NO levels.^43^ We found NO levels were below the levels of detection in infected MDMs, consistent with the recognised minor role of NO-mediated killing in human macrophages.^44^ Human MSCs can clear intracellular *M. tuberculosis* through autophagy in vitro^45^ though MSCs from patients with TB appear susceptible to bacterial subversion ex vivo.^46^ One phase 1 clinical trial has reported on autologous MSC therapy for TB,^15^ finding that a single dose was well tolerated as an adjunct therapy in patients with multidrug-resistant TB. There were no severe adverse effects or clinical deterioration associated with MSC infusion, nor in the following 6 months. MSC-treated patients had reduced CRP 1 month after MSC treatment, but the study was underpowered to assess for significance. One possible explanation is that endogenous MSCs are susceptible to mycobacteria, while exogenous human MSCs escape persistent infection through apoptosis or efferocytosis.^41^ More safety testing and dose-finding studies will be required to translate MSCs into further trials for TB and MAC-PD.

There were some limitations to this study. The sample size of macrophage donors in each experimental series was small (typically 4–6). Although these numbers were powered for significance and comparable with similar studies in the literature,^11 47^ the generalisability of our findings should be confirmed using a larger number of MDM donors from diverse backgrounds. Most of our work used BM-MSCs from one source, although we were able to reproduce similar immunomodulatory effects on intracellular bacterial replication in macrophages using MSCs from a second source. Nevertheless, differential therapeutic efficacy has been described between MSC sources and tissue types,^19 48^ highlighting the importance of testing MSCs from variable sources where feasible, and confirming whether the mechanism is consistent between them. This will be particularly beneficial for the optimisation and standardisation of MSC therapies for human trials. In the animal studies, we used human MSCs, which demonstrate greater antimicrobial efficacy and clinical relevance than murine MSCs,^26^ though mismatching species may also lose important molecular interactions. We did not examine for MSC effect on pathology, though mature granulomas are not typically evident in balb/c mice until 12 weeks post-infection^32^ and SARS-CoV-2 restrictions at our institution limited all chronic studies to 6 weeks. A larger and longer study with multiple endpoints to capture the immediate and long-term effects of treatments in established MAC-PD pathology would add further understanding to the efficacy of MSCs.^32^ This could be combined with pretreating MSCs and/or mice with celecoxib to see if the effect is COX-2 dependent in vivo. Additionally, MSCs should be explored as an adjunct therapy to antimicrobials in MAC-PD, particularly against clinical isolates of MAC which may have different growth and susceptibility patterns to laboratory reference strains. It is possible that concomitant antimicrobial use (including agents used in antimicrobial decontamination of in vitro media) could interfere with macrophage or MSC function.

In summary, MSCs can modulate inflammation and reduce intracellular MAC replication in human macrophages via a COX-2-PGE2-PI3K-dependent pathway and reduce bacterial proliferation in a murine model of chronic MAC-PD. These data support further assessment of MSCs as an adjunctive therapy for this resistant pathogen.

10.1136/thorax-2023-220819.supp2 Supplementary data

## Data Availability

Data are available upon reasonable request.
