# Supplementary material for: Human mesenchymal stromal cells inhibit Mycobacterium avium replication in clinically relevant models of lung infection
Source: Thorax. 2024 Mar 20;79(8):778–87. doi: 10.1136/thorax-2023-220819 (PMC11287638; doi:10.1136/thorax-2023-220819)
Supplement: Supplementary data [file thorax-2023-220819supp001.pdf]

Human mesenchymal stromal cells inhibit *Mycobacterium avium* replication in clinically-relevant models of lung infection

Timothy D Shaw, Anna D Krasnodembskaya, Gunnar N Schroeder, Declan F Doherty, Johnatas Dutra Silva, Shikha M Tandel, Yue Su, David Butler, Rebecca J Ingram, Cecilia M O'Kane

Online supplement

SUPPLEMENTARY MATERIALS AND METHODS

Bacterial culture

Mycobacterium avium subsp. *avium* Chester ATCC® 25291™ (American Type Culture Collection, Rockville, Maryland, USA) reference strain was cultured in Middlebrook 7H9 broth (BBL Becton Dickinson, USA), supplemented with 10% albumin-dextrose-catalase, 0.2% glycerol, 0.005% Tween 80, 100µg/ml ampicillin (Sigma-Aldrich, Burlington, Massachusetts, USA) and 10µg/ml Amphotericin B from *Streptomyces* sp. (Sigma-Aldrich). For confirmation studies, a clinical strain isolated from sputum of a patient with clinically-confirmed MAC pulmonary disease was donated by the Northern Ireland Mycobacterial Reference Laboratory (*M. avium* CI5). There were no notable difference in time for sub-cultures to reach mid-log phase between the strains.

The optical density (OD) of cultures was measured over time until attainment of log phase growth, confirmed by sequential colony counting at OD 600nm of 0.6-0.8, at which point they were used for cell infection. Bacteria were quantified by counting colony forming units per ml (CFU/ml) on serial 10-fold dilutions of specimen on Middlebrook 7H11 solid agar supplemented with 10% oleic acid-albumin-dextrose, 0.5% glycerol, 100µg/ml ampicillin (Sigma Aldrich) and 10µg/ml Amphotericin B from *Streptomyces* sp. (Sigma-Aldrich) after incubation at 37ºC, 5% CO_2 for 10-14 days.

Human monocyte-derived macrophage cell culture

Primary monocytes were sourced from healthy adult volunteers either from single donor leucocyte buffy coats (~50ml) donated by the Northern Ireland Blood

Shaw TD, *et al. Thorax* 2024; 79:778–787. doi: 10.1136/thorax-2023-220819

Transfusion Service, or from peripheral blood (50-100ml) donated through phlebotomy. Protocols for obtaining these blood products were approved by the Queen's University Belfast School Research Ethics Committee (Ref: 14.30v2; Title: Investigating regulation of inflammation and repair in Mycobacterial Infection).

Monocytes were isolated from blood donor residual buffy coat or peripheral blood by density gradient centrifugation across a Ficoll-Paque gradient (Sigma-Aldrich), seeded at $3x10^5$ per ml in Roswell Park Memorial Institute (RPMI) 1640 (Thermo Fisher, Waltham, Massachusetts, USA) supplemented with 1% heat-inactivated foetal bovine serum and 100µg/ml ampicillin (Sigma Aldrich) ($RPMI_{1\%FBS+AMP}$) and incubated at 37ºC, 5% CO_2 for 90 minutes. The wells were then washed once with Hanks' Balanced Salt Solution (HBSS) (Thermo Fisher) to remove non-adherent cells (erythrocytes, granulocytes and lymphocytes), leaving a monolayer of monocytes on the well surface. After washing, wells were replenished with $RPMI_{10\%FBS+AMP}$ supplemented with 10ng/ml recombinant granulocyte-macrophage colony stimulating factor (GM-CSF; Peprotech, Rocky Hill, New Jersey, USA) for 7 days at 37ºC, 5% CO_2 to promote differentiation into macrophages. MDMs were differentiated into alveolar-like macrophages using GM-CSF (Peprotech) to more closely model MAC infection of alveolar macrophages [S1,S2]. These MDMs have been previously confirmed to express M1-like markers (CD40+ and CD54+) with relative absence of M2-like markers (CD163+ and CD206+) on flow cytometry[S3]. After 7 days, the monocyte-derived macrophages were washed once in HBSS and incubated with $RPMI_{10\%FBS+AMP}$ for 24 hours prior to use in experiments.

Infection of MDMs

Shaw TD, *et al. Thorax* 2024; 79:778–787. doi: 10.1136/thorax-2023-220819

Monocyte-derived macrophages (3 x 10^5/ml) in a 24-well plate were washed once with HBSS and inoculated with mycobacterial culture to achieve MOI 1 in $RPMI_{1\%FBS+AMP}$. Plates were gently rocked to facilitate dispersal of bacteria before centrifugation at 280rcf, 20 ºC, for 5 minutes to promote bacterial-macrophage contact. After centrifugation, plates were incubated at 37ºC, 5% CO_2 for 4 hours. After the incubation period, supernatants were aspirated and discarded. Wells were washed three times with HBSS before replacement of media and addition of treatment conditions. Plates were then incubated at 37ºC, 5% CO_2 for up to 72 hours.

CFUs were enumerated for extracellular (supernatant) and intracellular (lysate) bacteria at specified time points. For lysates, cells were washed three times with HBSS before addition of 1ml lysis buffer (0.2% saponin in PBS) and incubation at 37ºC, 5% CO_2 for 30 minutes. Cells were then mechanically disrupted through scraping of the well surface. Cell suspensions were transferred into a fresh microtube, centrifuged at 12,000rcf for 5 minutes to remove cellular debris and bacteria, and transferred into a fresh microtube and stored at -80 ºC. The primary outcome measure used to calculate sample size was reduction in bacterial CFU. To demonstrate a conservative 20% reduction in CFU at 72 hours, with SD 0.1xmean, a minimum sample size of 4 per group was needed (80% power, at a $p<0.05$, two-way t-test).

Human bone-marrow derived mesenchymal stromal cell culture

Human bone marrow-derived MSCs (BM-MSCs) were purchased from ATCC (Product number PCS-500-012™) at passage 2 and expanded to a maximum of

Shaw TD, *et al. Thorax* 2024; 79:778–787. doi: 10.1136/thorax-2023-220819

passage 6. ATCC reported the MSCs were isolated from a healthy 24-year-old Caucasian male and characterised according to criteria stipulated by the International Society for Cellular Therapy (ISCT), including positive surface antigen expression for CD73, CD90 and CD105, and negative for CD45, CD34, CD14 and CD19 [S4]. In addition, these MSCs demonstrated differentiation capacity into adipocytes, chondrocytes and osteocytes. For confirmation studies, BM-MSCs from a second human donor were obtained from the Texas A&M Health Science Centre College of Medicine, Institute for Regenerative Medicine, USA which also met criteria stipulated by the ISCT as previously described[S5]. On receipt, MSCs were cultured in T175 culture flasks in α-Minimal Essential Medium (α-MEM) (Thermo Fisher) supplemented with 16.5% heat-inactivated fetal bovine serum, 1% L-glutamine and 100µg/ml ampicillin (α-$MEM_{16.5\%FBS+5\%LG+AMP}$) and incubated at 37ºC, 5% CO2, 21% O2.

MSCs were passaged on reaching 70% confluency. After removal of media, MSCs were washed once in HBSS and detached from the flask by adding 0.05% trypsin-EDTA (Thermo Fisher) and incubating for 5 minutes at 37ºC, 5% CO2. Detachment was also promoted mechanically by tapping of the culture flask. The trypsin was neutralised by adding an equal volume of α-$MEM_{16.5\%FBS+5\%LG+AMP}$. The cell solution was centrifuged at 285rcf for 5 minutes, with removal of the supernatant and resuspension in α-$MEM_{16.5\%FBS+5\%LG+AMP}$ prior to cell counting.

During treatment, MSCs were added directly to infected MDMs after washing at a ratio of 1 MSC : 3 MDMs in $RPMI_{1\%FBS+AMP}$. As MDMs were infected with MOI 1, this gave an overall cellular ratio of 1 MSCs : 3 MDMs : 3 MAC bacilli. Therefore, to keep

cell numbers consistent between comparable experiments, an MOI of 3 was during infections of MSCs alone (in the absence of MDMs). For transwell conditions, MSCs were added at the same ratio into transwell inserts with 0.4nm diameter pores (Merck Millipore, Burlington, Massachusetts, USA) to allow diffusion of soluble factors without cell-cell contact. Human pulmonary fibroblasts (CCD-11Lu cells, ATCC), cultured in identical media and conditions, were used as a stromal cell control.

During COX inhibition studies, MSCs were treated with celecoxib 2.5μM (Sigma-Aldrich, SML3031) or ibuprofen 100μmol/L (Sigma-Aldrich, PHR1004). MSCs were pre-treated with celecoxib at 2.5μM concentration for 30 minutes prior to their addition to transwells above MDMs. Celecoxib was also added to the transwells to sustain a starting concentration of 2.5μM during the MSC treatments. Celecoxib has an IC_{50} value of 0.04μM[S6] and concentrations degrade by up to 15% per 24 hours in tissue culture[S7]. Therefore we calculated a starting concentration of 2.5μM at t=0 would maintain levels above the IC_{50} value during a 72 hour experiment. Prostaglandin E2 (PGE2) 50ng/ml (Sigma-Aldrich, P0409) was used for stimulation studies. For phosphoinositide 3-kinase (PI3K) inhibition studies, MDMs were treated with wortmannin 1μM (Tocris, Bristol, UK, 1232) for 10 minutes before washing and treatment with MSCs.

Western blot analysis

Western blot analysis was performed as previously described[S8]. MSC-treated macrophages were lysed using radioimmunoprecipitation assay (RIPA) buffer supplemented with cOmplete EDTA-free protease inhibitor (Sigma Aldrich) and the

phosphatase inhibitor PhosSTOP (Sigma Aldrich). Protein concentration was determined using BCA protein assay (Micro BCA protein assay kit, ThermoFisher). 40µg of total protein of each sample was separated on 10% SDS-PAGE under reducing conditions and transferred onto a PVDF membrane (GE Healthcare, Freiberg, Germany). The membranes were blocked with 5% milk and incubated with rabbit anti-human primary phospho-Akt (Ser473) antibody (1:1000, #9271, Cell Signalling; Danvers, Massachusetts, USA) overnight at 4° C. After washing, the blots were incubated with HRP-conjugated anti-rabbit IgG secondary antibody (1:1000, Cell Signalling) for 2h at RT. Protein expression was visualised using SuperSignal™ West Femto Maximum Sensitivity Substrate (ThermoFisher) at G:Box Chemi imaging system (Syngene; Cambridge, UK). Membranes were stripped using Restore™ Western Blot Stripping Buffer (Thermo Fisher) and re-probed with rabbit anti-human primary Akt antibody (1:1000, Cell Signalling, #9272) before re-visualisation.

Animal studies

Balb/c mice have previously been validated as an immunocompetent animal model of chronic MAC-PD for testing antimicrobial therapies [S9]. Six-week-old female balb/c mice (Charles River, Alderley Park, UK) were maintained in cages in the Biological Services Unit at Queen's University Belfast, following a 7-day acclimatisation period. Experiments were reviewed and approved by the UK Home Office and QUB Ethical Review Committee and performed in accordance with ARRIVE (Animal Research: Reporting of *In Vivo* Experiments) guidelines and the Declaration of Helsinki conventions for the use and care of animals. During experiments, mice were checked and weighed twice weekly. Any mice found to have breached the severity limit were culled by a schedule 1 method (cervical dislocation)

immediately and were excluded from analysis. The primary outcome measure used to calculate sample size was reduction in pulmonary CFU. To detect a presumed mean 40% reduction in pulmonary CFU, with SD 0.3xmean, a minimum sample size of 9 per group was needed (80% power at $p<0.05$, two-way t-test).

M. avium Chester strain in 50ml of 7H9 broth at OD 0.6-0.8 was centrifuged at 1000rcf and resuspended in sterile PBS at 10^6 or 10^9 CFU/ml. An aerosol chamber was created by re-purposing a Pennock-style plethysmography double-chamber (EMMS, Bordon, UK), originally designed to study pulmonary function tests in mice exposed to nebulised compounds. Mice were placed in the chamber (approx. 200ml volume) and exposed to nebulised *M. avium* at 50% airspace density for 5 minutes. The nebuliser head was cleaned and inoculum replaced after each cage group was infected (every five mice) to prevent bacterial clogging. On day 5 post-infection, five mice were sacrificed to check pulmonary infection had been established. Lungs were homogenised in 1ml of sterile PBS and plated in serial 10-fold dilutions onto 7H11 agar for colony counting. To reduce confounding factors, infected mice were caged in groups of five and randomised within each cage by a blinded investigator (D.F.D.) to receive either 1 million human BM-MSCs in 100µl PBS or vehicle control (100µl PBS) via tail vein injection (29G needle) on days 21 and 28 p.i.. One investigator (T.D.S.) remained aware of group allocation and treatment outcome throughout the study. Tail vein injections are technically challenging and mice were excluded from analysis if administration of therapy failed for either dose, for example, if there was extravasation of the injected bolus into tail or accidental loss of bolus during handling. At the study end point, mice were culled by anaesthetic overdose via 140mg/kg sodium pentobarbital administered by intraperitoneal injection. Lungs,

spleen and liver were harvested, homogenised in 1ml of sterile PBS (Precellys® bead homogenisation, Berlin, Germany) and plated in serial 10-fold dilutions onto 7H11 agar for colony counting. CFUs were counted blinded to treatment group. Lung homogenate was then clarified by centrifugation and aspiration of supernatant for protein analysis.

Two experiment replicates were performed – the first with n=5 per group, followed by a larger study with n=8 in the MSC treatment group and n=6 in the placebo group. Both experiments included an uninfected control group, n=3 in the first study and n=5 in the second. In the second study, administration of MSC tail vein injections failed in two mice, which were excluded from analysis. Data from remaining mice from both experimental replicates were combined for analysis (total n=11 per treatment group and n=8 for uninfected control group).

Shaw TD, *et al. Thorax* 2024; 79:778–787. doi: 10.1136/thorax-2023-220819

SUPPLEMENTARY FIGURES

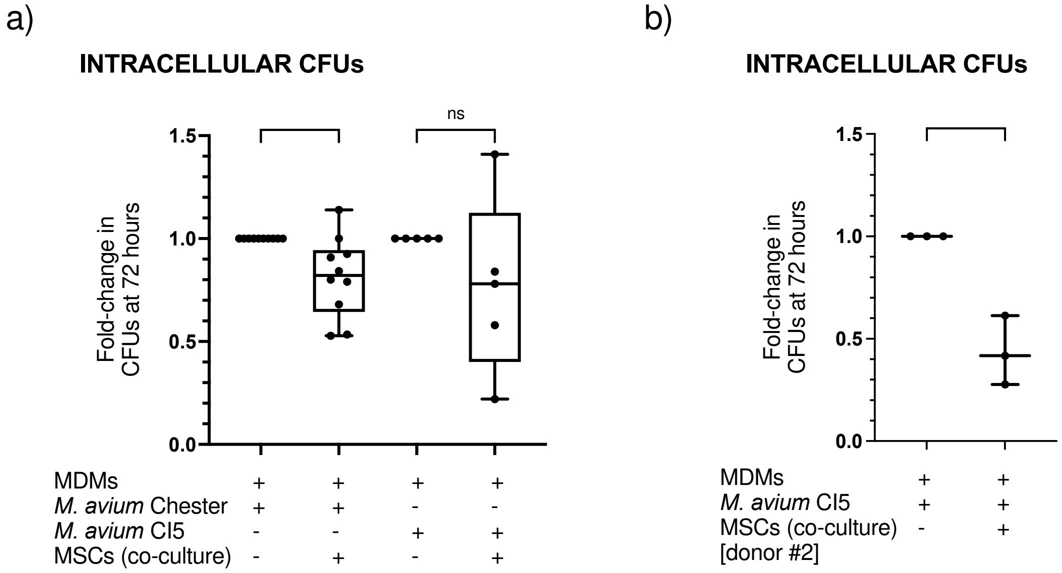

Figure S1: MSC inhibition of intracellular *M. avium* is not strain- or donor-specific

MSCs conferred an overall fall in intracellular CFUs in MDMs from 10 healthy volunteers infected with *M. avium* Chester ($p<0.05$) and 5 healthy volunteers infected with clinical isolate CI5, though this did not quite meet statistical significance ($p=0.08$) (a). However, the antimicrobial effect of MSCs on *M. avium* CI5 was confirmed using MSCs from a second donor ($p<0.01$) (b). Data presented as median with IQR and analysed using the Kruskal-Wallis test with Dunn's multiple comparison test (a, n=5-10) or unpaired t-test (b, n=3).

Shaw TD, *et al. Thorax* 2024; 79:778–787. doi: 10.1136/thorax-2023-220819

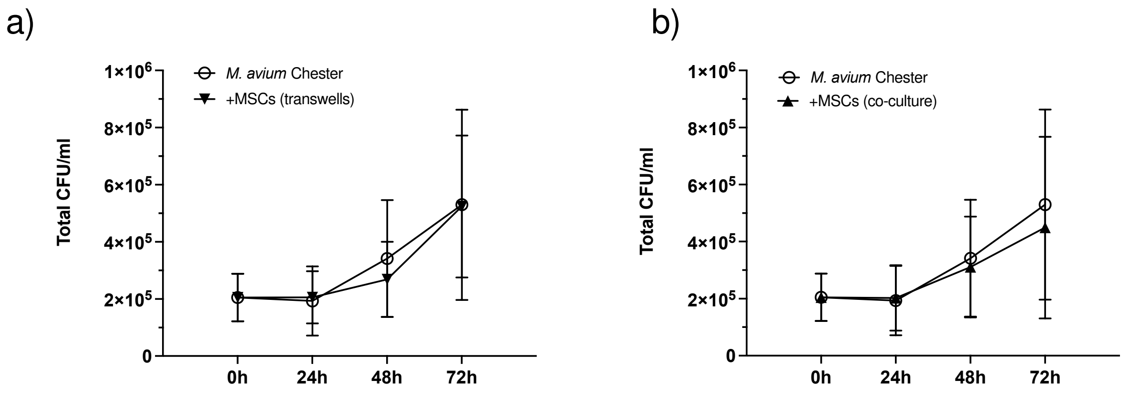

Figure S2: Direct effect of MSCs on the growth of *M. avium* over 72 hours

Total CFU increased over time in the presence and absence of MSCs in transwells (a) and direct co-culture (b) over 72 hours ($p<0.05$) with no difference in bacterial viability between groups. Data presented as mean + SD and analysed by two-way ANOVA. N=4.

Shaw TD, *et al. Thorax* 2024; 79:778–787. doi: 10.1136/thorax-2023-220819

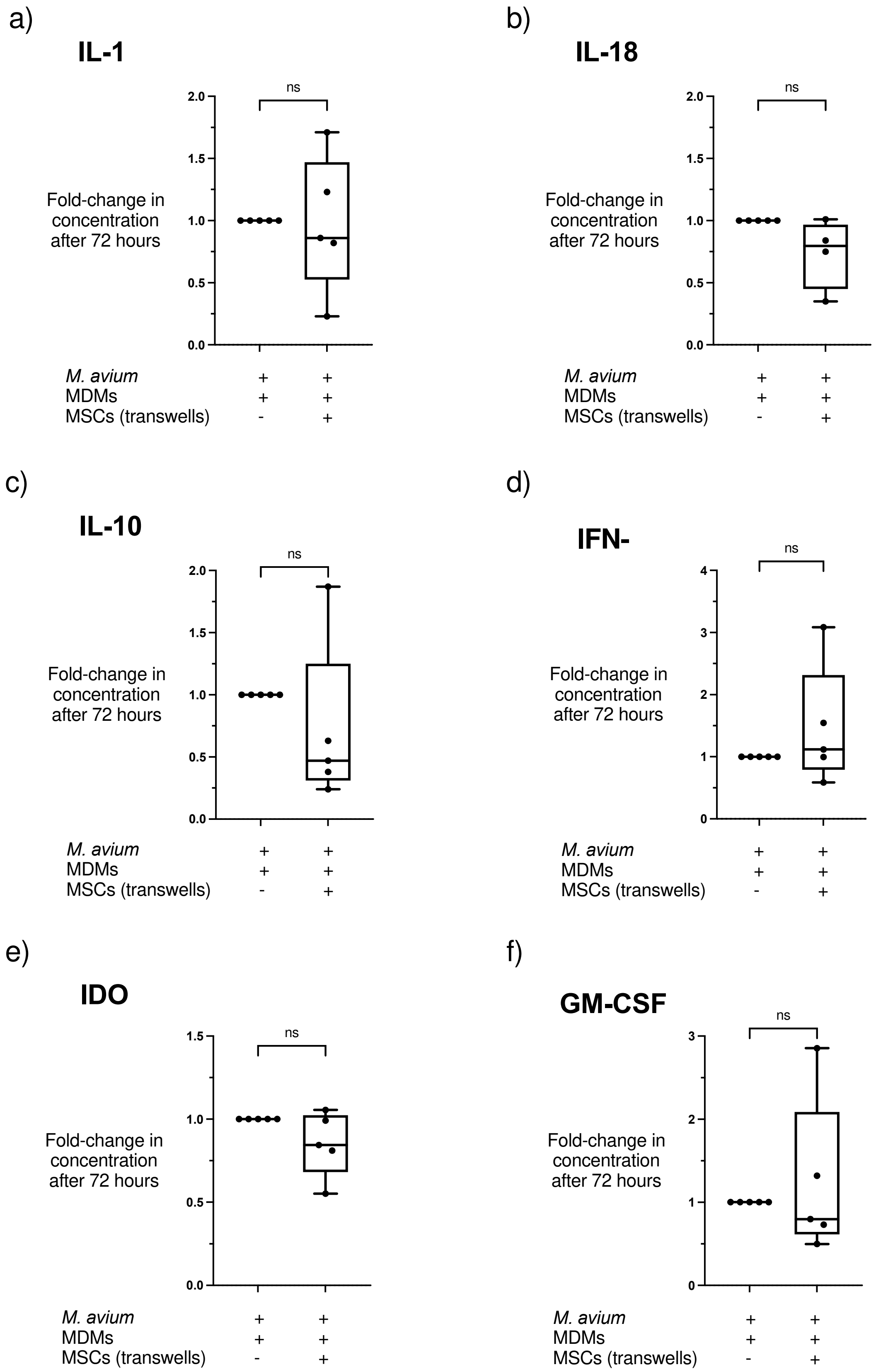
a)
IL-1
ns
Fold-change in concentration after 72 hours
0.0
0.5
1.0
1.5
2.0
M. avium + +
MDMs + +
MSCs (transwells) - +
b)
IL-18
ns
Fold-change in concentration after 72 hours
0.0
0.5
1.0
1.5
2.0
M. avium + +
MDMs + +
MSCs (transwells) - +
c)
IL-10
ns
Fold-change in concentration after 72 hours
0.0
0.5
1.0
1.5
2.0
M. avium + +
MDMs + +
MSCs (transwells) - +
d)
IFN-
ns
Fold-change in concentration after 72 hours
0
1
2
3
4
M. avium + +
MDMs + +
MSCs (transwells) - +
e)
IDO
ns
Fold-change in concentration after 72 hours
0.0
0.5
1.0
1.5
M. avium + +
MDMs + +
MSCs (transwells) - +
f)
GM-CSF
ns
Fold-change in concentration after 72 hours
0
1
2
3
M. avium + +
MDMs + +
MSCs (transwells) - +

Figure S3: the effect of MSCs on levels of important mediators of macrophage function

The levels of other important mediators of macrophage activation against intracellular mycobacteria were measured in MDMs treated with MSCs in transwells. There were trends towards reduced levels of IL-1β (A), IL-18 (B) and IL-10 (C), but these did not reach significance. Neither did MSCs mediate a significant change in levels of IFN-γ (D) IDO (E) or GM-CSF (F) in the presence of infected MDMs. Data presented as median with IQR and analysed using the Mann-Whitney test. N=4-5; ns = not significant.

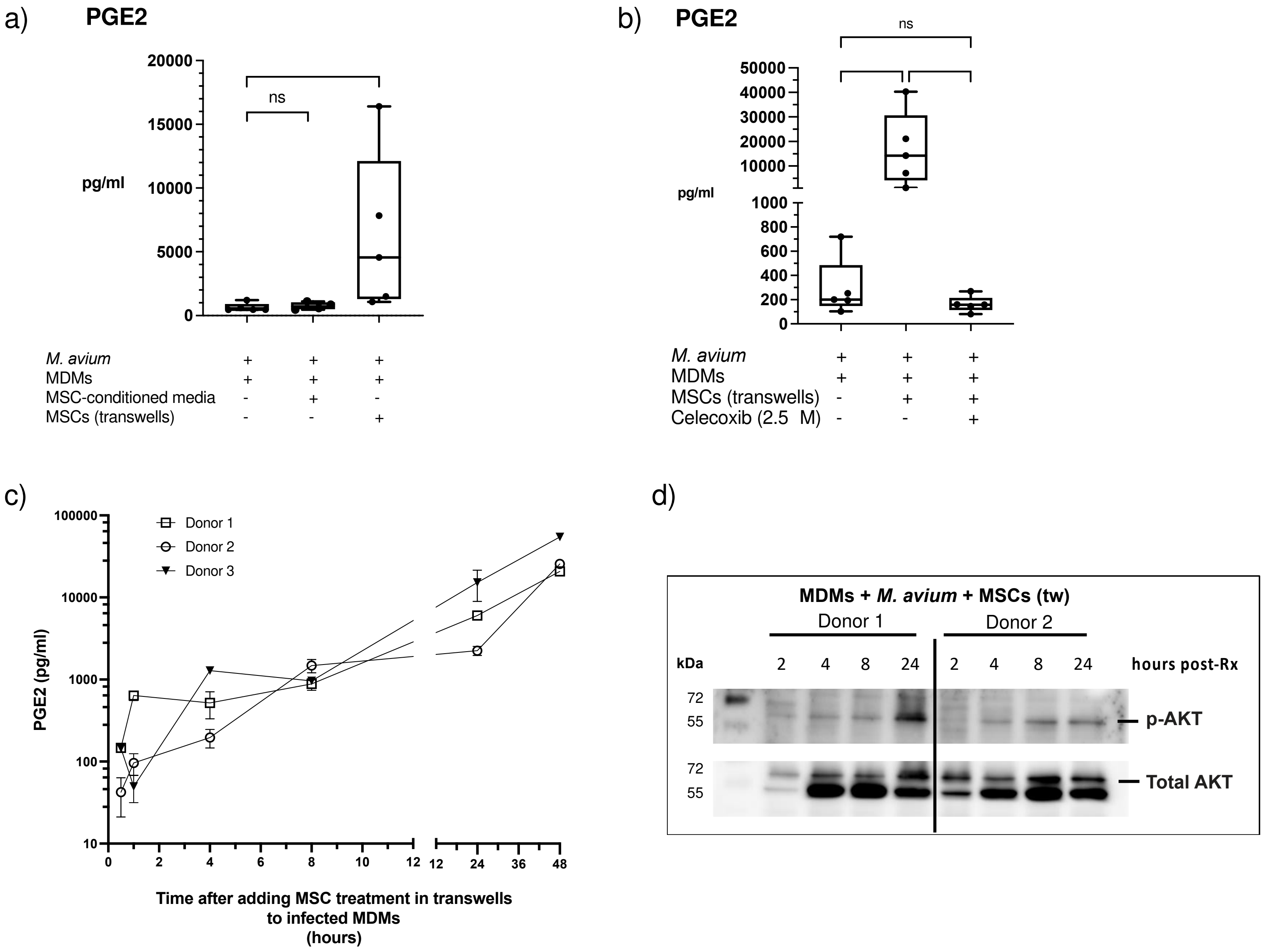
a)
PGE2
pg/ml
ns
M. avium
MDMs
MSC-conditioned media
MSCs (transwells)
b)
PGE2
pg/ml
ns
M. avium
MDMs
MSCs (transwells)
Celecoxib (2.5 M)
c)
Donor 1
Donor 2
Donor 3
PGE2 (pg/ml)
Time after adding MSC treatment in transwells to infected MDMs (hours)
d)
MDMs + *M. avium* + MSCs (tw)
Donor 1
Donor 2
kDa
hours post-Rx
p-AKT
Total AKT

Figure S4: The differential MSC-derived PGE2 response between MDM donors

PGE2 concentrations rose when MAC-infected MDMs were treated with MSCs, but not with MSC-conditioned media, after 72 hours (a). The increased concentration of PGE2 mediated by MSCs was abrogated in the presence of celecoxib ($p<0.05$) (b). However, the rate and volume of PGE2 secreted during MSC treatment of infected MDMs varied between macrophage donors (c). By 8 hours post-treatment, PGE2 concentration had reached approximately 1000pg/ml for all three donors and continued to rise over 48 hours. Phosphorylated AKT in MSC-treated infected MDMs was detectable after 4 hours, with increased levels at 8 and 24 hours (n=2 donors) (d). Data presented as median with IQR and analysed using the Kruskal-Wallis test with Dunn's multiple comparison test. N=5 (a+b) * $p < 0.05$; ns = not significant.

Shaw TD, *et al. Thorax* 2024; 79:778–787. doi: 10.1136/thorax-2023-220819

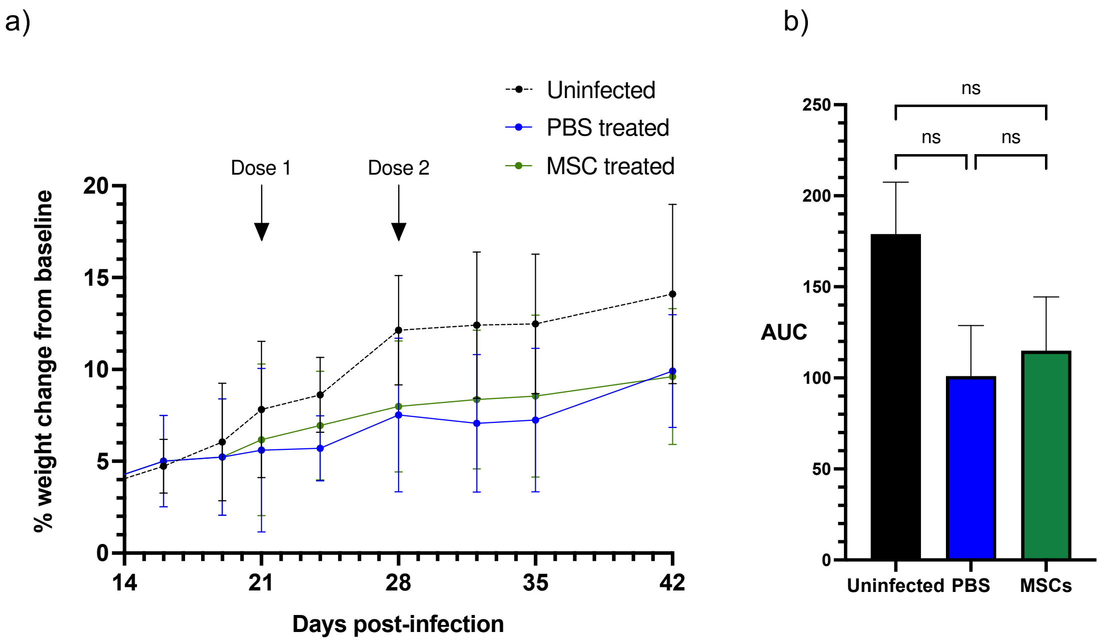

Figure S5: Effect of MSC treatment on weight gain

Over 42 days, uninfected mice had gained more weight than infected mice treated with placebo and MSC-treated mice (a), though this did not reach statistical significance (p=0.15 and 0.28 respectively) (b). There was also no difference in weight gain between placebo- and MSC-treated mice (p=0.93).

Data presented as mean with standard deviation and groups were analysed by two-way ANOVA (a). AUC presented as mean with standard error of the mean and analysed by unpaired t-test (b). N=8 for uninfected group, n=11 for PBS-treated, n=11 for MSC-treated and data combined from two independent experiments.

Shaw TD, *et al. Thorax* 2024; 79:778–787. doi: 10.1136/thorax-2023-220819

SUPPLEMENTARY DATA TABLES

Table S1: Raw data for Figure 1

MDMs + M.avium						
	Donor	1	2	3	4	5
Extracellular CFUs (supernatant)	0h	-	-	-	-	-
	72h	6.78E+05	1.79E+04	4.33E+04	9.78E+04	9.33E+04
Intracellular CFUs (lysate)	0h	1.94E+06	1.42E+05	3.09E+05	1.80E+06	6.23E+05
	72h	5.99E+06	1.96E+05	2.38E+05	1.11E+06	6.76E+05
Total CFUs (combined)	0h	1.94E+06	1.42E+05	3.09E+05	1.80E+06	6.23E+05
	72h	6.67E+06	2.13E+05	2.81E+05	1.21E+06	7.69E+05
+ MSCs (co-culture)						
	Donor	1	2	3	4	5
Extracellular CFUs (supernatant)	0h	-	-	-	-	-
	72h	1.17E+06	1.97E+04	2.86E+04	5.44E+04	1.01E+05
Intracellular CFUs (lysate)	0h	1.94E+06	1.42E+05	3.09E+05	1.80E+06	6.23E+05
	72h	2.39E+06	1.60E+05	1.20E+05	1.04E+06	6.10E+05
Total CFUs (combined)	0h	1.94E+06	1.42E+05	3.09E+05	1.80E+06	6.23E+05
	72h	3.56E+06	1.80E+05	1.49E+05	1.10E+06	7.11E+05
**Fold-change from MDMs+M.avium**						
**72h IC fold-change**		**0.40**	**0.82**	**0.50**	**0.94**	**0.90**
**72h total fold-change**		**0.53**	**0.84**	**0.53**	**0.91**	**0.92**
+ MSCs (transwells)						
	Donor	1	2	3	4	5
Extracellular CFUs (supernatant)	0h	-	-	-	-	-
	72h	8.89E+05	4.37E+04	3.51E+04	1.12E+05	1.06E+05
Intracellular CFUs (lysate)	0h	1.94E+06	1.42E+05	3.09E+05	1.80E+06	6.23E+05
	72h	3.88E+06	1.06E+05	1.10E+05	7.56E+05	6.46E+05
Total CFUs (combined)	0h	1.94E+06	1.42E+05	3.09E+05	1.80E+06	6.23E+05
	72h	4.77E+06	1.49E+05	1.45E+05	8.68E+05	7.51E+05
**Fold-change from MDMs+M.avium**						
**72h IC fold-change**		**0.65**	**0.54**	**0.46**	**0.68**	**0.96**
**72h total fold-change**		**0.71**	**0.70**	**0.52**	**0.72**	**0.98**
+ Fibroblasts (co-culture)						
	Donor	1	2	3	4	5
Extracellular CFUs (supernatant)	0h	-	-	-	-	-
	72h	9.78E+05	4.22E+04	1.61E+04	N/A	8.44E+04
Intracellular CFUs (lysate)	0h	1.94E+06	1.42E+05	3.09E+05	N/A	6.23E+05
	72h	5.27E+06	2.66E+05	2.00E+05	N/A	7.23E+05
Total CFUs (combined)	0h	1.94E+06	1.42E+05	3.09E+05	N/A	6.23E+05
	72h	6.25E+06	3.08E+05	2.16E+05	N/A	8.08E+05
**Fold-change from MDMs+M.avium**						
**72h IC fold-change**		**0.88**	**1.36**	**0.84**	N/A	**1.07**
**72h total fold-change**		**0.94**	**1.44**	**0.77**	**N/A**	**1.05**

CFUs, colony-forming units; MDMs, monocyte-derived macrophages

Shaw TD, *et al. Thorax* 2024; 79:778–787. doi: 10.1136/thorax-2023-220819

Table S2: Raw data for Figure 5

	Day 5 lung CFUs		Day 42 lung CFUs		Day 42 spleen CFUs	
**Innoculum**	**10^6 CFU/ml**	**10^9 CFU/ml**	**10^6 CFU/ml**	**10^9 CFU/ml**	**10^6 CFU/ml**	**10^9 CFU/ml**
**Mouse 1**	2.70E+04	7.77E+04	3.33E+07	7.00E+06	1.63E+05	3.00E+04
**Mouse 2**	1.97E+04	6.83E+04	3.20E+07	6.77E+06	2.33E+05	1.77E+04
**Mouse 3**	2.33E+04	6.20E+04	2.50E+07	7.43E+06	2.43E+05	5.30E+04
**Mouse 4**	1.70E+04	9.00E+04	2.30E+07	3.90E+06	2.90E+05	1.43E+04
**Mouse 5**	2.63E+04	6.93E+04	2.60E+07	2.93E+06	2.07E+05	9.50E+03
**Mouse 6**	-	-	1.33E+07	-	1.10E+05	-
**Mouse 7**	-	-	6.00E+06	-	2.63E+04	-
**Mouse 8**	-	-	1.13E+07	-	7.53E+04	-
**Mouse 9**	-	-	4.33E+06	-	7.67E+04	-
**Mouse 10**	-	-	1.33E+07	-	4.17E+04	-
**25% Percentile**	1.84E+04	9.98E+06	9.98E+06	3.42E+06	6.69E+04	1.19E+04
**Median**	2.33E+04	1.82E+07	1.82E+07	6.77E+06	1.37E+05	1.77E+04
**75% Percentile**	2.67E+04	2.75E+07	2.75E+07	7.22E+06	2.36E+05	4.15E+04
**Mean**	2.27E+04	7.35E+04	1.88E+07	5.61E+06	1.47E+05	2.49E+04
**Std. Deviation**	4.28E+03	1.08E+04	1.04E+07	2.04E+06	9.31E+04	1.74E+04

CFUs, colony-forming units

Shaw TD, *et al. Thorax* 2024; 79:778–787. doi: 10.1136/thorax-2023-220819

SUPPLEMENTARY REFERENCES

S1 Portevin D, Gagneux S, Comas I, *et al.* Human macrophage responses to clinical isolates from the Mycobacterium tuberculosis complex discriminate between ancient and modern lineages. *PLoS Pathog*. 2011;7:e1001307-.

S2 Susanta P, Eusondia A, Jan S, *et al.* A new tractable method for generating human alveolar macrophage-like cells in vitro to study lung inflammatory processes and diseases. *mBio*. 2023;14:e00834-23.

S3 Morrison TJ, Jackson M V, Cunningham EK, *et al.* Mesenchymal stromal cells modulate macrophages in clinically relevant lung injury models by extracellular vesicle mitochondrial transfer. *Am J Respir Crit Care Med*. 2017;196:1275–86.

S4 Dominici M, Le Blanc K, Mueller I, *et al.* Minimal criteria for defining multipotent mesenchymal stromal cells. The International Society for Cellular Therapy position statement. *Cytotherapy*. 2006;8:315–7.

S5 Jackson M V, Morrison TJ, Doherty DF, *et al.* Mitochondrial Transfer via Tunneling Nanotubes is an Important Mechanism by Which Mesenchymal Stem Cells Enhance Macrophage Phagocytosis in the In Vitro and In Vivo Models of ARDS. *Stem Cells*. 2016;34:2210–23.

S6 Torcris. Celecoxib: certificate of analysis. 2021.

S7 Mendes M, Sousa J, Pais A, *et al.* Can Celecoxib Assay in Preclinical Studies Be Improved? Processes. 2023;11. https://doi.org/10.3390/pr11020431

S8 Dutra Silva J, Su Y, Calfee CS, *et al.* Mesenchymal stromal cell extracellular vesicles rescue mitochondrial dysfunction and improve barrier integrity in clinically relevant models of ARDS. *European Respiratory Journal*. 2021;58:2002978.

S9 Andréjak C, Almeida D V., Tyagi S, *et al.* Characterization of mouse models of Mycobacterium avium complex infection and evaluation of drug combinations. *Antimicrob Agents Chemother*. 2015;59:2129–35.
