# Supplementary material for: Human mesenchymal stromal cells inhibit Mycobacterium avium replication in clinically relevant models of lung infection
Source: Thorax. 2024 Mar 20;79(8):778–87. doi: 10.1136/thorax-2023-220819 (PMC11287638; doi:10.1136/thorax-2023-220819)
Supplement: Supplementary data [file thorax-2023-220819supp002.pdf]

BMJ Publishing Group Limited (BMJ) disclaims all liability and responsibility arising from any reliance placed on this supplemental material which has been supplied by the author(s)

Human mesenchymal stromal cells inhibit *Mycobacterium avium* replication in clinically-relevant models of lung infection

Shaw TD, et al. *Thorax* 2024. DOI: 10.1136/thorax-2023-220819

Mesenchymal stromal cells (MSCs) caused PGE2-dependent reduction in *M. avium* colony-forming units (CFU) in human monocyte-derived macrophages (MDMs)

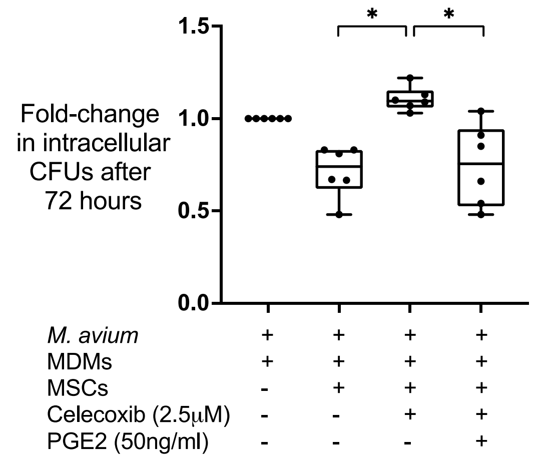

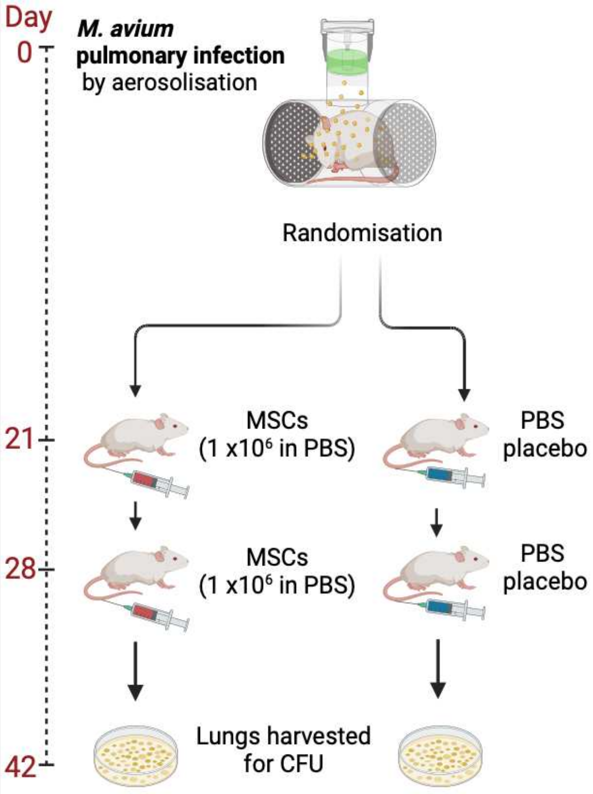

MSCs showed a similar antimicrobial effect in an *in vivo* model of *M. avium* complex pulmonary disease.

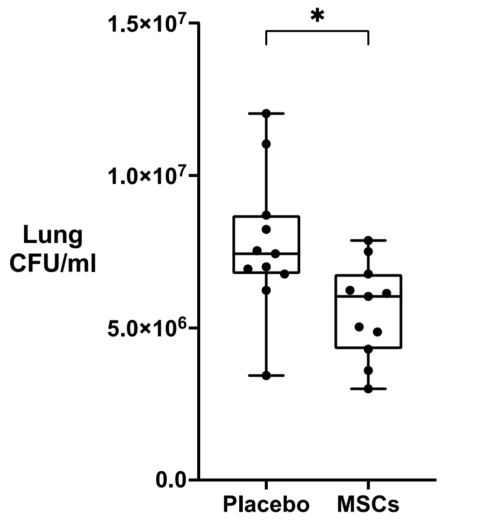

Thorax thorax.bmj.com @ThoraxBMJ British Thoracic Society BMJ
